# Sex-Specific Difference in the Effect of Altitude on Sleep and Nocturnal Breathing in Young Healthy Volunteers

**DOI:** 10.3390/jcm11102869

**Published:** 2022-05-19

**Authors:** Taomei Li, Lu Tan, Michael Furian, Yanyan Zhang, Lian Luo, Fei Lei, Xiaofang Xue, Jiaming He, Xiangdong Tang

**Affiliations:** 1 Sleep Medicine Center, Mental Health Center, Department of Respiratory and Critical Care Medicine, State Key Laboratory of Biotherapy, West China Hospital, Sichuan University, Chengdu 610041, China; litaomei@scu.edu.cn (T.L.); tanlu2022@scu.edu.cn (L.T.); zhyanyan1314520@163.com (Y.Z.); luolian2022@wchscu.cn (L.L.); leifei525@wchscu.cn (F.L.); 2 Sleep Disorders Center, Department of Respiratory Medicine, University Hospital of Zurich, 8091 Zurich, Switzerland; michael.furian@usz.ch; 3 Department of Cardiology, Diqing Tibetan Autonomous Prefectural People’s Hospital, Shangri-La 674400, China; xuexfaa@163.com (X.X.); hejm_2022@outlook.com (J.H.)

**Keywords:** sleep-disordered breathing, altitude, sex differences, apnea–hypopnea index, acute mountain sickness

## Abstract

Importance: To date, there is no established evidence of sex-specific differences in altitude-induced sleep-disordered breathing (SDB) during polysomnography-confirmed sleep. Objective: The aim of this study was to investigate whether differences in sex play a pivotal role in incidences of SDB and acute mountain sickness (AMS) when staying overnight at high altitude. Design: This was a prospective cohort study. Setting: Participants underwent overnight polysomnography (PSG) and clinical assessment in a sleep laboratory at 500 m and two consecutive days at 3270 m. Participants: The participants comprised 28 (18 women) healthy, young, low-altitude residents with a median (interquartile range) age of 26.0 (25.0, 28.0) years. Exposures: Altitude exposure. Main outcomes and Measures: The primary outcome was altitude-induced change in the PSG-confirmed apnea–hypopnea index (AHI) at 3270 m compared to 500 m between men and women. Secondary outcomes included sex differences in other parameters related to SDB, sleep structure, AMS, psychomotor vigilance test reaction time and parameters from arterial and venous blood analyses. Results: The median (interquartile range) AHIs at 500 m and 3270 m on night 1 and on night 2 were 6.5/h (3.6, 9.1), 23.7/h (16.2, 42.5) and 15.2/h (11.8, 20.9) in men, respectively, and 2.2/h (1.0, 5.5), 8.0/h (5.3, 17.0) and 7.1/h (4.9, 11.5) in women, respectively (*p* < 0.05 nights 1 and 2 at 3270 m vs. 500 m in men and women). The median difference (95% CI) of altitude-induced change in AHI (3270 m night 1 compared to 500 m) between men and women was 11.2/h (1.9 to 19.6) (*p* < 0.05). Over the time course of 2 days at 3270 m, 9 out of 18 (50%) women and 1 out of 10 (10%) men developed AMS (*p* < 0.05 women versus men). Conclusions and Relevance: This prospective cohort study showed that men were more susceptible to altitude-induced SDB but that they had a lower AMS incidence when staying for 2 days at 3270 m than women. These findings indicate that sex-related prevention and intervention strategies against SDB and AMS are highly warranted. Trial Registration: This trial was registered at the Chinese Clinical Trial Registry; No. ChiCTR1800020155.

## 1. Introduction

Mountain tourism has become increasingly popular in recent years and accounts for 15–20% of global tourism, which represents USD 70–90 billion per year [1,2]. When travelling to high altitude, barometric and oxygen partial pressures decrease, and altitude-dependent hypoxemia occurs [3]. Moreover, hypoxemia has been shown to be associated with altitude-induced sleep-disordered breathing (SDB), sleep fragmentation and acute mountain sickness (AMS), three conditions reducing well-being and daytime performance when staying at high altitude [4,5,6,7].

Sleep disturbances are common complaints among lowlanders ascending to high altitude and may result in higher proportions of superficial sleep, reduced subjective sleep quality and decreased cognitive performance [4,8]. One underlying cause of impaired sleep at high altitude is high-altitude periodic breathing characterized by an oscillatory pattern of waxing and waning of ventilation with periods of hyperventilation alternating with central apneas and hypopneas [9,10]. This pattern of SDB has been shown to be associated with more arousals, less deep sleep (non-rapid eye movement (NREM) sleep stage 3) and lower subjective sleep quality [5,11]. 

Sex-specific differences in the susceptibility to high-altitude periodic breathing and sleep fragmentation are not well understood. At least at low altitude, SDB, especially, obstructive sleep apnea, has long been considered a predominantly male-related disease [12,13] and has been shown to be exacerbated at high altitude [14,15]. This was confirmed in native male highlanders, having higher AHI and a higher percentage of severe OSA than female highlanders [16]. These findings suggest that men rather than women might be at risk for SDB at high altitude; however, women remain highly underrepresented in high-altitude studies. In accordance with this, the majority of previous high-altitude studies assessing SDB and sleep structure included predominantly male participants aged 30-45 years [9,17,18]. One study at 4559 m reported less SDB in women compared to men; unfortunately, sleep structure was not assessed [19]. 

Additionally, AMS, characterized by headache, nausea, fatigue and sleep disturbance, is the most commonly seen high-altitude illness. AMS affects more than 25% of individuals ascending to 3500 m and more than 50% of those reaching elevations above 6000 m [7]. The main risk factors for the development of AMS are the speed of ascent and individual AMS susceptibility [20]. Previous studies reported high [21] or no difference [22,23] in AMS susceptibility in women compared to men. In 2019, the authors of an AMS meta-analysis concluded that women are more prone to develop AMS compared to men [24]; however, simultaneously, the definition of AMS, often assessed using the Lake Louise questionnaire, has been amended, and the occurrence of sleep disturbances is no longer part of the AMS definition [25].

Therefore, the purpose of this study was to quantify sex-specific differences in sleep-disordered breathing and AMS incidence during a 2-day sojourn to high altitude. Based on preliminary findings from previous altitude studies, we hypothesized that altitude-induced SDB will be higher in men than in women but that the AMS incidence will be higher in women than in men. 

## 2. Material and Methods

### 2.1. Participants

Healthy, young, Chinese volunteers living <800 m, who were aged between 18 and 30 years, without any chronic cardiorespiratory disease and without regular medication (including medications affecting respiratory center drive) were invited to take part in this study. Exclusion criteria were smoking (>1 pack/day), regular alcohol intake, altitude exposure above 1500 m in the last 6 months for more than 1 week and a baseline apnea–hypopnea index (AHI) greater than 15 events/h. 

### 2.2. Study Design and Setting

This prospective cohort study was carried out from August 2017 to November 2018. Participants completed their baseline evaluation at Chengdu (500 m, barometric pressure (PB) of 718 mmHg) and ascended thereafter within one month by airplane (about 1.5 h) to Shangri-La (3270 m, PB of 488 mmHg). Altitude evaluations were performed in the sleep laboratory of Diqing Tibetan Autonomous Prefectural People’s Hospital. During the stay at altitude, participants rested in a quiet hotel nearby and were free in their activities; however, strenuous exercise, caffeinated drinks and alcohol were discouraged. The study was approved by the West China Hospital of Sichuan University Biomedical Research Ethics Committee (Ethic number: 2016(90)), and participants gave their written informed consent. The trial was registered in the Chinese Clinical Trial Registry (registration number ChiCTR1800020155).

### 2.3. Measurements 

#### 2.3.1. Clinical Examination and Questionnaires

A detailed medical history, a clinical evaluation, a lung function assessment and questionnaires were obtained during the screening period. The Epworth sleepiness scale (ESS) [26] was used to assess excessive daytime sleepiness; the Pittsburgh sleep quality index (PSQI) [27] was used to assess sleep quality and other potential sleep-related symptoms; the 7-item generalized anxiety disorder scale (GAD-7) [28] and the 9-item patient health questionnaire (PHQ-9) [29] were used to assess anxiety and depression symptoms. The Mallampati score was also obtained during the screening period for each participant as previously described [30]. Morning questionnaires, including self-reported sleep latency, total sleep time and wake periods after sleep onset, were used to evaluate subjective sleep quality. Supine blood pressure (BP) was measured (DS-1902, Nissei, Gunma, Japan) before and after the sleep study. Spirometry (forced vital capacity (FVC) and forced expiratory volume in the first second of expiration (FEV_1_)) (MasterScreen PET System; Jaeger, Germany) were performed according to international guidelines [31].

#### 2.3.2. Sleep Studies

All participants underwent overnight polysomnography (PSG) (Alice 6; Respironics Inc., Murrysville, PA, USA) at 500 m and in the first two consecutive nights at 3270 m. PSG was performed and scored according to the American Academy of Sleep Medicine (AASM) guidelines (version 2.0) [32]. The recordings included electroencephalography, bilateral electrooculography, electrocardiography, electromyography (submental and anterior tibialis), nasal and oral thermal airflow, nasal pressure, thoraco-abdominal movements and pulse oximetry. Sleep stages, arousals and respiratory events were scored by investigators blinded to the clinical data and study location. An apnea was defined as more than 90% reduction in airflow for at least 10 s. Obstructive apnea was differentiated from central apnea by continued or increased inspiratory effort throughout the entire period of absent airflow. Mixed apneas were scored as obstructive events in this study to be consistent with our previous study [33]. Hypopnea was defined as a 30% or more reduction in nasal pressure for at least 10 s, associated with at least 3% reduction in oxygen saturation or arousal. Obstructive hypopnea was differentiated from central hypopnea when there was snoring, an associated thoraco-abdominal paradox or increased inspiratory flattening of the nasal pressure compared to baseline breathing. Oxygen desaturation was defined as an at least 3% reduction in oxygen saturation. Total, central and obstructive AHIs were computed as the sum of corresponding apneic and hypopneic events divided by total sleep time. 

#### 2.3.3. Daytime Evaluation

The symptoms and severity of acute mountain sickness (AMS) were assessed using the self-reported Lake Louise questionnaire (LLS) throughout the 2-day stay at high altitude. The participants were considered to have AMS if their LLS score was ≥3 points covering a headache score ≥ 1 [34]. Because the LLS was revised in 2018 and sleep disturbances are no longer part of AMS, we performed post hoc analyses to calculate AMS defined by the 2018 LLS [25]. 

In the morning, a 10 min psychomotor vigilance test (PVT) [35] was performed in a silent room. Participants were instructed to concentrate on the screen and press the “space” key as soon as possible when a red dot appeared on the screen. A PVT response was regarded valid if the reaction time (RT) was ≥100 ms. Lapses (errors of omission) were defined as RTs ≥ 500 ms. The mean RT, fastest 10% RT, slowest 10% RT and number of lapses were assessed and included in the analyses.

#### 2.3.4. Blood Tests

Venous blood samples were drawn in the morning after overnight PSG recordings. Red and white blood cell counts, platelet counts, hematocrit level and hemoglobin concentration were measured using an automated hematology analyzer (Sysmex XN-9000; Sysmex, Kobe, Japan). Arterial blood gas samples were drawn from the radial artery while the subjects rested quietly in supine position to obtain pH, PaO_2_ and PaCO_2_.

### 2.4. Outcomes

The primary outcome of this study was the altitude-induced change in AHI at 3270 m compared to 500 m between men and women. Secondary outcomes included sex differences in other parameters related to SDB, sleep structure, AMS, PVT reaction time and parameters from arterial and venous blood analyses. No a priori sample size estimation was performed.

### 2.5. Statistical Analysis

Due to the non-normal distribution of the majority of the outcomes, data are presented as medians (interquartile range (IQR)) for continuous variables and as number and proportions for categorical variables. The effect of altitude (3270 m vs. 500 m) and acclimation (day 2 vs. day 1 at 3270 m) were evaluated using the Friedman analysis of variance. If the Friedman ANOVA showed an overall significance, paired comparisons were performed using Wilcoxon matched pairs tests. Chi-square or Fisher’s Exact test was used to compare the differences in categorical data. The median difference and 95% CI between high and low altitude and between women and men were calculated using the Hodges–Lehmann estimate. To assess predictors for SDB at 3270 m, univariate and multivariate least-square regression models were performed, using total or central AHI as a dependent variable and altitude (3270 m vs. 500 m), baseline characteristics (age, body mass index, sex, Mallampti score, AHI), days spent at altitude, AMS score, pH and PaCO_2_ as independent variables. Total and central AHIs were log-transformed to obtain a normal distribution. Predictor variables with a probability of *p* < 0.2 in the univariate analysis were entered into a subsequent multivariate model. Statistical significance was assumed when *p* < 0.05 or 95% CI of mean differences did not overlap zero. SPSS 22.0 was used for data analysis.

## 3. Results 

### 3.1. Participants

A total of 37 participants were invited and assessed for eligibility. One participant was excluded because of smoking and regular alcohol intake. Two participants were excluded because of ascending to altitude > 1500 m during the past 6 months. Four participants were further excluded because their AHIs were over 15/h at 500 m, and two participants withdrew their consent after baseline examinations. Thus, a total of 28 (18 women) participants were included in the final analysis. Two female participants suffered from severe AMS (LLS scores of 8 and 9) on the second day at 3270 m and received oxygen treatment. They did not accomplish PSG assessments during night 2 or daytime evaluations the day after (Figure S1).

The median (IQR) age and BMI were 27.5 years (24.8, 29.5) and 23.3 kg/m^2^ (20.6, 26.7) in men, respectively, and 26.0 years (24.3, 27.0) and 21.3 kg/m^2^ (20.0, 24.5) in women, respectively (*p* > 0.05 for both comparisons). There were no significant differences in other demographic parameters between female and male participants, except for women having lower neck and waist and hip circumferences than men (Table 1). 

### 3.2. Respiratory Events, Oxygen Saturations, Heart Rate and Blood Pressure

Sleep studies at 500 m revealed a normal nocturnal breathing pattern in men and women with median (IQR) AHIs of 6.5 events/h (3.6, 9.1) and 2.2 events/h (1.0, 5.5) (*p* < 0.05) and mean oxygen saturations (SpO_2_) of 96.0% (95.0, 97.0) and 96.0% (95.0, 97.0), respectively (*p* > 0.05 between women and men). During the first night at 3270 m, AHI significantly increased to 23.7 events/h (16.2, 42.5) in men and to 8.0 events/h (5.3, 17.0) in women, with predominant increases in central events (Table 2, Figure S2). The mean SpO_2_ decreased to medians of 79.0% (75.5, 84.5) and 82.0% (81.0, 85.0) in men and women, respectively. The second night at altitude revealed persistent elevations in AHI and ODI.

**Figure 1:**
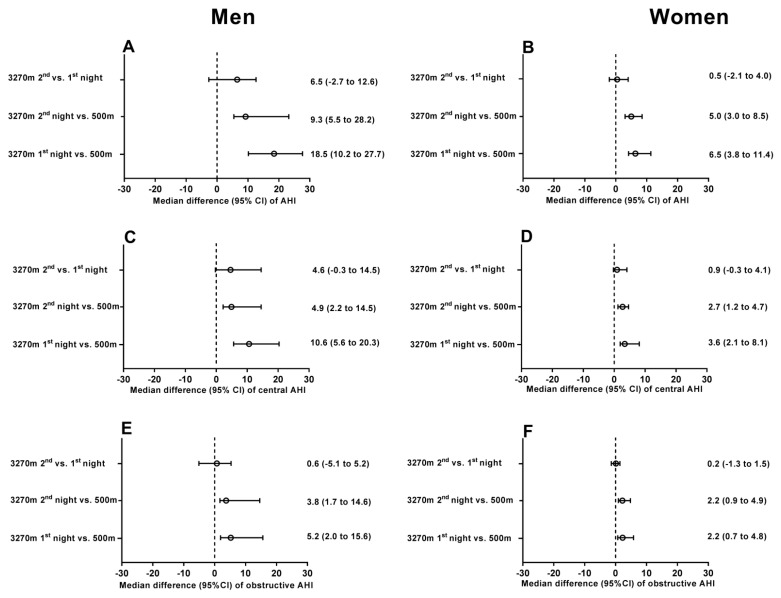
Median difference (95% CI) in total AHI (Panels **A** and **B**), central AHI (Panels **C** and **D**) and obstructive AHI (Panels **E** and **F**) with altitude ascent in men and women. AHI, apnea–hypopnea index.

Correspondingly, the altitude-induced median changes (95% CI) in total AHI in men and women were 18.5 events/h (10.2 to 27.7) and 6.4 events/h (4.2 to 11.4), respectively, and they were significantly higher in men than in women by a median difference (95% CI) of 11.2 events/h (1.9 to 19.6, *p* < 0.05) (Table 3 and Figure 1). The higher increase in total AHI in men was mainly caused by an increase in central events. Correspondingly, the time with periodic breathing and the ODI increased more in men than in women when travelling to 3270 m (Table 3); however, the mean nocturnal oxygen saturation changed similarly with altitude in women and men.

The multivariate regression models showed that altitude and male sex were significant predictors of total and central AHIs (Table 4), even after correcting for age, BMI and other confounders. Moreover, baseline AHI at 500 m was positively associated with total AHI, and it was marginally associated with central AHI at 3270 m (*p* = 0.050, Table 4).

Objective and subjective sleep parameters are presented in Table S1. No significant differences were found in sleep structure, sleep efficiency or subjective sleep characteristics between altitudes and between women compared to men. The altitude induced an increase in heart rate during rapid eye movement (REM) sleep and non-REM sleep in both women and men. Evening systolic and diastolic blood pressures were elevated in male participants at 3270 m, while no effect of altitude was observed in women.

### 3.3. Clinical and Daytime Evaluations

As shown in Table S2, eight (28.6%; seven women and one man, *p* = 0.194 between women and men) and five (19.2%; four women and one man, *p* = 0.617 between women and men) participants met the criteria for AMS on the first and the second days at altitude, respectively. Over the time course of both days, 9 out of 18 (50%) women compared to 1 out of 10 (10%) men suffered from AMS (*p* < 0.05 between women and men), while 8 out of 18 (44.4%) women and 1 out of 10 (10%) men had AMS described in the 2018 LLS. The psychomotor vigilance test performances expressed by the mean RT and fastest 10% RT were prolonged in women, while no changes were observed in men with altitude ascent (Table S2). Arterial blood gas analysis revealed an elevation in pH and decreases in PaCO_2_ and PaO_2_ with altitude in men and women alike, while men had lower SaO_2_ and PaO_2_ values during the second day at 3270 m. Red blood cell count, hematocrit level, white blood cell count and platelet count increased in women at altitude, while only hematocrit levels increased in men (Table S2).

The association between AMS severity and female sex was marginally non-significant, whereas high altitude was predictive for prolonged reaction time derived from PVT independent of sex (Table S3).

## 4. Discussion

This prospective cohort study in healthy, young, lowland participants revealed sex-specific differences in SDB and clinical outcomes, such as AMS incidence, psychomotor vigilance performance and blood pressure, during acute exposure to an altitude of 3270 m. We found considerable elevations in AHIs in both men and women with altitude ascent, and they were mainly caused by an increase in central events. The AHI and the altitude-induced change in AHI were significantly elevated in men compared to women, while women were more prone to develop AMS. Psychomotor vigilance performance was impaired in women, while altitude induced an increase in blood pressure in men.

The effects of acute altitude exposure on sleep and breathing in healthy low-altitude dwellers have previously been studied. Our results on the effect of altitude are consistent with those of previous studies showing an increase in AHI due to an increase in central events [5,9]. Apart from a significant increase in central events, obstructive events increased in men and women, which is consistent with what Tseng et al. previously reported in healthy participants ascending to 3150 m (a change in obstructive events from 0.4 ± 0.2/h at sea level to 2.5 ± 2.7/h at 3150 m) [36]. The observed association between total AHI (mainly characterized by obstructive events) and the Mallampati score indicates that the propensity for upper airway collapse and the unstable control of breathing may lead to more respiratory events at altitude. During the second night, total AHI decreased compared to the first night at 3270 m, suggesting partial acclimatization.

We found that men had more altitude-induced SDB than women when ascending to 3270 m, which was previously indicated by a clinical trial investigating the effect of sex and acetazolamide on periodic breathing at high altitude. Furthermore, in a study conducted by Caravati et al., men (*n* = 11, 39.3 ± 10.7 years) showed a higher AHI than women (*n* = 10, 35.2 ± 9.5 years) in the placebo arm (40.9 ± 27.8/h vs. 7.3 ± 4.0/h, *p* = 0.008) [19]. Another study conducted by Lombardi et al. confirmed the sex-related differences in nocturnal breathing patterns at high altitude [37]; however, both of the cited studies did not perform polysomnographic measurements, and, therefore, no information about sleep structure or AHI was available. Therefore, to the best of our knowledge, the current study is the first to investigate sex-specific differences in altitude-induced SDB during polysomnography-confirmed sleep. Furthermore, we showed that sex remained an independent predictor for AHI at 3270 m when controlling for baseline confounders. The possible underlying mechanisms for the sex-related difference in AHI at high altitude were not investigated in the current study and remain elusive. However, sex-related differences in the hypoxic ventilatory response [38], baseline AHI and differences in upper airway anatomy [39,40] might have contributed to the observed sex-related differences. Although we did not measure airway length or pharyngeal volume in our study, men had a higher neck circumference, indicating more soft tissue. Moreover, men may have had a higher apnea threshold and a lower CO_2_ reserve [41], therefore predisposing them to more nocturnal breathing instabilities compared to women.

In our study, the incidence of AMS on the second morning at altitude was 28.6%, which is in accordance with the expected AMS incidence at this altitude. However, only a few studies have reported AMS separately in women and men; therefore, the lack of findings hampers comparisons. During the 2-day sojourn to 3270 m, women had a higher AMS incidence than men (50% vs. 10%, *p* < 0.05). Moreover, the multivariate regression analysis revealed that not only the incidence but also the AMS severity was marginally higher in women than in men. Our findings confirm the previous conclusions from a meta-analysis investigating the relationship between AMS and sex [24]. Moreover, in our post hoc analysis, we found that the morbidity of AMS was 9/28 (32.1%), which is in line with the morbidity described in the old definition.

Regarding the changes in objective and subjective sleep, we found no significant differences in sleep structure or subjective sleep quality in this young group during the first two nights at 3270 m. These surprising results are in contrast to those of previous studies demonstrating reduced slow wave sleep and subjective quality, and impaired sleep continuity with increases in the arousal index and wake time after sleep onset [4,5]. The inconsistent findings may be related to differences in ethnicity and the ascent protocol, due to the younger age (median age of 26 years) of our subjects or due to the lack of statistical power.

The daytime assessments revealed altitude-induced impairments in cognitive performance in women but not in men and that cognitive impairment was associated with lower mean nocturnal SpO_2_ but not with SDB. In contrast, blood pressure mainly increased in men and not in women; however, sex differences in blood pressure regulation at high altitude require further investigations.

This prospective cohort study provides important insights into sex-related differences in altitude-induced changes in SDB, sleep, AMS, daytime outcomes and cognitive performance. However, the reported findings represent acute altitude effects and might not represent conditions during prolonged exposure. Additionally, this study was not able to investigate underlying mechanisms explaining sex-related differences in nocturnal breathing, i.e., the hypoxic ventilatory response, apnea threshold or CO_2_ reserve, and no information about menstrual cycle phases or oral contraceptives was assessed in women.

## 5. Conclusions

In this prospective cohort study, we found higher altitude-induced AHI and blood pressure but lower AMS incidence and less severe cognitive impairments in men than in women during acute exposure to 3270 m. These findings pave the way to investigate sex-specific prevention and treatment strategies against SDB and AMS in young healthy men and women staying overnight at high altitude.

## Figures and Tables

**Table 1:** Demographic and clinical characteristics of male and female participants.

	Men (*n* = 10)	Women (*n* = 18)	*p* Value
Numbers	10	18	
Age, y	27.5 (24.8, 29.5)	26.0 (24.3, 27.0)	0.245
BMI, kg/m^2^	23.3 (20.6, 26.7)	21.3 (20.0, 24.5)	0.356
Neck circumference, cm	36.5 (35.5, 38.3)	31.5 (30.8, 32.9)	<0.001
Waist circumference, cm	84.0 (78.5, 93.5)	71.8 (69.0, 82.8)	0.020
Hip circumference, cm	101.0 (93.8, 106.5)	92.0 (90.0, 93.3)	0.010
Tonsil hypertrophy, *n* (%)	1 (10.0)	2 (11.1)	0.716
Tongue hypertrophy, *n* (%)	2 (20.0)	2 (11.1)	0.452
Mallampati score			0.826
Class IV, *n* (%)	0	0	
Class III, *n* (%)	1 (10.0)	1 (5.6)	
Class II, *n* (%)	1 (10.0)	3 (16.7)	
Class I, *n* (%)	8 (80.0)	14 (77.8)	
FEV_1_, L	3.8 (3.4, 4.2)	3.0 (2.9, 3.3)	0.002
FEV_1_, % predicted	98 (92, 101)	105 (95, 108)	0.106
FVC, L	4.8 (3.7, 5.0)	3.5 (3.1, 3.7)	0.011
FVC, % predicted	98 (90, 113)	101 (97, 109)	0.787
FEV_1/_FVC	82.1 (76.2, 86.2)	86.0 (82.8, 94.2)	0.106
ESS	5.5 (3.8, 9.3)	5.5 (2.0, 8.8)	0.494
PSQI	2.5 (0.5, 4.0)	2.5 (1.8, 4.0)	0.834
GAD-7	0.5 (0, 4.8)	1.0 (0.8, 2.5)	0.697
PHQ-9	2.0 (1.0, 6.8)	4.0 (0.8, 6.0)	0.976

Median (interquartile range) for continuous variables, and *n* (%) for category variables. BMI, body mass index; FEV_1_, forced expiratory volume in the first second of expiration; FVC, forced vital capacity; ESS, Epworth sleepiness scale; PSQI, Pittsburgh sleep quality index; GAD-7, 7-item generalized anxiety disorder scale; PHQ-9, 9-item patient health questionnaire.

**Table 2:** Nocturnal breathing parameters and blood pressure in men and women at 500 m and 3270 m.

	Men				Women			
	500 m	3270 m 1st Night	3270 m 2nd Night	ANOVA, *p* Value	500 m	3270 m 1st Night	3270 m 2nd Night	ANOVA, *p* Value
**Sleep Breathing Parameters**								
Total AHI/h	6.5 (3.6, 9.1) *	23.7 (16.2, 42.5) ^a,^*	15.2 (11.8, 20.9) ^a,^*	0.002	2.2 (1.0, 5.5)	8.0 (5.3, 17.0) ^a^	7.1 (4.9, 11.5) ^a^	<0.001
Central AHI/h	0.6 (0.3, 2.6)	11.7 (7.3, 19.1) ^a,^*	7.0 (2.8, 9.6) ^a^	<0.001	0.4 (0.1, 0.9)	4.0 (1.9, 7.1) ^a^	2.7 (1.2, 5.3) ^a^	<0.001
Central apnea index/h	0.6 (0.3, 2.6)	5.1 (3.0, 11.3) ^a,^*	3.5 (2.0, 6.7) ^a,^*	0.014	0.4 (0.1, 0.9)	1.5 (0.7, 2.6) ^a^	1.2 (0.5, 2.0) ^a^	0.029
Central hypopnea index/h	0 (0, 0)	4.9 (3.3, 10.3) ^a^	1.6 (1.0, 5.4) ^a,b^	<0.001	0 (0, 0)	2.0 (0.6, 5.8) ^a^	1.3 (0.5, 3.3) ^a^	<0.001
Mean CAH duration, s	13.5 (12.0, 15.2)	14.1 (13.7, 15.0)	13.4 (12.9, 14.4)	0.690	13.8 (12.0, 14.7)	14.3 (13.0, 16.4)	14.2 (12.8, 15.0)	0.256
Obstructive AHI/h	4.3 (2.5, 7.4) *	9.0 (6.0, 22.8) ^a,^*	8.8 (4.7, 16.0) ^a,^*	0.008	1.8 (0.9, 3.8)	3.8 (2.2, 7.6)^a^	3.6 (2.1, 7.8) ^a^	0.022
Obstructive apnea index/h	0.2 (0, 0.5)	0 (0, 1.2)	0.1 (0, 0.3)	0.508	0 (0.1, 0.4)	0 (0, 0.2)	0 (0.1, 0.3)	0.695
Obstructive hypopnea index/h	3.6 (2.2, 7.2) *	8.1 (6.0, 21.5) ^a,^*	8.7 (4.7, 15.7) ^a,^*	0.002	1.8 (0.8, 3.2)	3.6 (1.9, 7.2)^a^	3.6 (1.8, 6.6) ^a^	0.009
Mean OAH duration, s	26.9 (23.6, 30.4) *	18.0 (16.5, 19.6) ^a^	17.8 (16.0, 19.2) ^a^	0.001	20.4 (17.9, 24.0)	16.5 (15.2, 17.6) ^a^	16.3 (15.3, 17.1) ^a^	0.003
PB duration, %TST	0 (0, 0)	0.9 (0.2, 2.8) ^a,^*	0 (0, 1.1)	0.002	0 (0, 0)	0 (0, 0.2)	0 (0, 0)	0.350
Oxygen desaturation index/h	4.6 (2.1, 10.4)	35.5 (31.2, 50.8) ^a,^*	24.9 (16.8, 36.7) ^a,^*	<0.001	2.7 (0.9, 4.7)	17.7 (11.9, 26.5) ^a^	12.5 (9.5, 18.4) ^a^	<0.001
Mean oxygen saturation, %	96.0 (95.0, 97.0)	79.0 (75.5, 84.5) ^a^	83.0 (73.2, 85.3) ^a^	<0.001	96.0 (95.0, 97.0)	82.0 (81.0, 85.0) ^a^	83.5 (79.5, 85.8) ^a^	<0.001
SpO_2_ < 85%, %TST	0 (0, 0)	95.6 (45.5, 98.6) ^a^	74.5 (41.8, 97.5) ^a^	<0.001	0 (0, 0)	85.3 (41.1, 95.7) ^a^	74.1 (42.1, 96.7) ^a^	<0.001
**Blood Pressure**								
Evening systolic blood pressure, mmHg	119 (110, 122) *	126 (123, 133) ^a,^*	121.5 (121.0, 126.8) ^a,b,^*	0.003	106 (98, 111)	98 (96, 113)	107 (100, 109)	0.089
Evening diastolic blood pressure, mmHg	77 (66, 82)	79 (75, 85) ^a,^*	79.5 (71.3, 86.3) ^a,^*	0.018	69 (59, 75)	65 (56, 77)	71 (64, 78)	0.111
Morning systolic blood pressure, mmHg	118 (112, 119) *	114 (110, 125) *	117.0 (112.0, 129.0) *	0.625	100 (971, 109)	103 (98, 111)	103 (99, 107)	0.943
Morning diastolic blood pressure, mmHg	77 (69, 79) *	76 (72, 83) *	76.0 (64.5, 83.0)	0.889	64 (58, 71)	66 (64, 75)	69 (61, 79)	0.576

Values presented as median (interquartile range). AHI, apnea–hypopnea index; CAH, central apnea hypopnea; OAH, obstructive apnea hypopnea; PB, periodic breathing; TST, total sleep time; SpO_2_, arterial oxygen saturation derived from pulse oximetry. ^a^ *p* < 0.05 comparing with 500 m within the same sex; ^b^ *p* < 0.05 comparing with 3270 m 1st night within the same sex; * *p* < 0.05 men vs. women at identical altitude and day.

**Table 3:** Altitude-induced changes in respiratory events and oxygen saturation during the first night.

	Median Difference (95% CI) between First Night at 3270 m vs. 500 m in Men	Median Difference (95% CI) between First Night at 3270 m vs. 500 m in Women	Median Difference (95% CI) of Altitude-Induced Changes in Men vs. Women	*p*
AHI/h	18.5 (10.2 to 27.7)	6.5 (3.8 to 11.4)	11.2 (1.9 to 19.6)	0.020
Central AHI/h	10.6 (5.6 to 20.3)	3.6 (2.1 to 8.1)	6.6 (0.1 to 10.3)	0.049
Central apnea index/h	4.4 (1.9 to 11.3)	1.1 (0.4 to 2.3)	3.1 (0.1 to 5.6)	0.049
Central hypopnea index/h	5.9 (3.4 to 9.0)	2.7 (1.3 to 5.6)	2.8 (0 to 5.1)	0.055
Mean CAH duration, s	0.7 (−1.0 to 2.4)	0.2 (−1.2 to 1.7)	0.7 (−1.6 to 3.0)	0.336
Obstructive AHI/h	5.2 (2.0 to 15.6)	2.2 (0.7 to 4.8)	2.6 (−0.3 to 6.7)	0.055
Obstructive apnea index/h	0 (−0.5 to 1.0)	0 (−0.2 to 0.2)	0 (−0.3 to 0.7)	0.661
Obstructive hypopnea index/h	5.3 (1.6 to 16.4)	2.3 (0.6 to 5.0)	2.2 (−1.0 to 7.0)	0.231
Mean OAH duration, s	−9.0 (−11.3 to −7.0)	−4.4 (−7.1 to −1.6)	−4.6 (−8.0 to −1.2)	0.010
PB duration, %TST	1.1 (0.3 to 4.8)	0 (0 to 0.3)	0.6 (0.1 to 1.5)	0.006
Oxygen desaturation index/h	32.6 (24.6 to 44.2)	16.8 (12.4 to 22.3)	15.6 (7.1 to 25.1)	0.003
Mean oxygen saturation, %	−16.0 (−20.5 to −11.0)	−13.5 (−15.0 to −12.0)	−3.0 (−7.0 to 2)	0.238
SpO_2_ < 85%, %TST	77.8 (52.2 to 98.0)	70.9 (49.5 to 91.3)	3.7 (−21.9 to 23.2)	0.415

Values represent median differences (95% CI). AHI, apnea–hypopnea index; CAH, central apnea hypopnea; OAH, obstructive apnea hypopnea; PB, periodic breathing; %TST, percentage of total sleep time; SpO_2_, arterial oxygen saturation derived from pulse oximetry.

**Table 4:** Univariate and multivariate regression models investigating predictors for total and central apnea–hypopnea indexes.

	Total AHI				Central AHI			
	Univariate Analysis		Multivariate Analysis		Univariate Analysis		Multivariate Analysis	
Independent Variables	Coefficient (95% CI)	*p*	Coefficient (95% CI)	*p*	Coefficient (95% CI)	*p*	Coefficient (95% CI)	*p*
Age, years	0.01 (−0.03, 0.04)	0.794			−0.03 (−0.09, 0.03)	0.329		
BMI, kg/m^2^	0.02 (−0.01, 0.05)	0.118	−0.02 (−0.03, 0.001)	0.063	−0.02 (−0.06, 0.03)	0.497		
Sex (men vs. women)	0.38 (0.20, 0.56)	<0.001	0.18 (0.06, 0.30)	0.004	0.38 (0.08, 0.68)	0.013	0.16 (0.56, 0.04)	0.026
Mallampati score								
III vs. I	0.25 (−0.26, 0.75)	0.340			−0.17 (−0.94, 0.61)	0.668		
II vs. I	0.27 (0.03, 0.52)	0.027	0.28 (0.14, 0.42)	<0.001	0.09 (−0.29, 0.47)	0.624		
pH	5.95 (3.23, 8.68)	<0.001	2.62 (0.70, 4.54)	0.008	9.47 (5.40, 13.53)	<0.001	3.98 (−0.96, 7.41)	0.128
PaCO_2_, mmHg	0.00 (−0.03, 0.02)	0.859			−0.01 (−0.05, 0.03)	0.579		
AMS, LLS score	0.03 (−0.03, 0.09)	0.323			0.09 (0.01, 0.18)	0.035	−0.01 (−0.09, 0.07)	0.784
Baseline AHI/h	0.06 (0.04, 0.08)	<0.001	0.06 (0.04, 0.07)	<0.001	0.05 (0.02, 0.09)	0.007	0.03 (0.00, 0.06)	0.050
Altitude (3270 m vs. 500 m)	0.52 (0.35, 0.69)	<0.001	0.49 (0.34, 0.64)	0.004	0.95 (0.71, 1.20)	<0.001	0.94 (0.56, 1.31)	<0.001
Days at altitude (2nd vs. 1st)	−0.24 (−0.13, −0.35)	<0.001	−0.15 (−0.27, −0.02)	0.022	0.30 (−0.01, 0.61)	0.059	−0.21 (−0.48, 0.07)	0.150

BMI, body mass index; PaCO_2_, arterial partial pressure of carbon dioxide; AMS, acute mountainous sickness assessed using the Lake Louise questionnaire (LLS); AHI, apnea–hypopnea index. Total and central AHIs were log-transformed to obtain a normal distribution.

## Data Availability

Data is available upon request to the corresponding author.

## Supplementary Materials

The following supporting information can be downloaded at: https://www.mdpi.com/article/10.3390/jcm11102869/s1, Table S1. Sleep parameters in men and women at 500 m and 3270 m. Table S2. Daytime evaluations in women and men at 500 m and 3270 m. Table S3. Univariate and multivariate generalized least square regression analysis models investigating predictors for AMS score and PVT. Figure S1. Flowchart. Figure S2. Individual values in total AHI (Panel A and B), central AHI (Panel C and D) and obstructive AHI (Panel E and F) at 500 m and 3270 m during the first and second night in men and women.

## Abbreviations

AHI	Apnea–hypopnea index
AMS	Acute mountain sickness
BMI	Body mass index
CSA	Central sleep apnea
ESS	Epworth sleepiness scale
FEV_1_	Forced expiratory volume in the first second of expiration
FVC	Forced vital capacity
GAD-7	7-item generalized anxiety disorder scale
OSA	Obstructive sleep apnea
PHQ-9	9-item patient health questionnaire
PSG	Polysomnography
PSQI	Pittsburgh sleep quality index
PVT	Psychomotor vigilance test
SDB	Sleep-disordered breathing
