# Supplementary material for: Sex-Specific Difference in the Effect of Altitude on Sleep and Nocturnal Breathing in Young Healthy Volunteers
Source: J Clin Med. 2022 May 19;11(10):2869. doi: 10.3390/jcm11102869 (PMC9143383; doi:10.3390/jcm11102869)
Supplement: Supplementary file 1 [file jcm-11-02869-s001.zip › jcm-1669327-supplementary.pdf]

Sex-specific difference in the effect of altitude on sleep and nocturnal breathing in young healthy volunteers

(Supplemental material)

Taomei Li[1,†], Lu Tan[1,†], Michael Furian [2], Yanyan Zhang [1], Lian Luo [1], Fei Lei [1], Xiaofang Xue [3], Jiaming He [3], Xiangdong Tang [1,*]

1 Sleep Medicine Center, Mental Health Center, Department of Respiratory and Critical Care Medicine, State Key Laboratory of Biotherapy, West China Hospital, Sichuan University, 610041 Chengdu, China

2 Sleep Disorders Center, Department of Respiratory Medicine, University Hospital of Zurich, 8091 Zurich, Switzerland

3 Department of Cardiology, Diqing Tibetan Autonomous Prefectural People's Hospital, 674400 Shangri-La, China

† These authors make equal contributions to this study.

*** Corresponding Author:**

Xiangdong Tang

Sleep Medicine Center, West China Hospital, Sichuan University

28 Dian Xin Nan Jie, Chengdu, Sichuan 610041, China

Tel: 86-28-85422733

Fax: 86-28-85422632

Email: 2372564613@qq.com/ tangxiangdong@scu.edu.cn

Table S1 Sleep parameters in men and women at 500 m and 3270 m

	Men				Women			
	**500 m**	**3270 m 1st night**	**3270 m 2nd night**	**ANOVA, P value**	500 m	3270 m 1st night	3270 m 2nd night	**ANOVA, P value**
**Objective sleep**								
Sleep latency, min	7.8 (4.0, 26.3)	7.3 (3.0, 15.8)	5.8 (3.8, 8.9)	0.500	13.0 (6.8, 21.4)	9.5 (5.3, 15.5)	9.8 (5.1, 18.4)	0.229
TST, min	426.9 (393.9, 468.0)	418.5 (368.8, 458.6)	449.5 (400.3, 457.4)	0.407	427.3 (359.3, 460.7)	441.5 (396.0, 465.9)	451.8 (426.3, 473.0)	0.269
NREM 1, %	14.1 (11.2, 17.0)	19.3 (13.6, 20.3)	12.9 (9.9, 15.8)	0.058	10.7 (8.3, 17.2)	13.1 (8.6, 17.6)	10.4 (7.4, 12.4)	0.444
NREM 2, %	50.3 (48.1, 53.1)	48.9 (41.7, 51.1)	49.2 (44.3, 50.7)	0.301	49.5 (45.1, 58.6)	49.0 (44.5, 54.0)	48.7 (45.0, 53.8)	0.444
NREM 3, %	14.5 (11.3, 15.5)	16.1 (10.3, 19.1)	16.0 (13.5, 17.6)	0.407	13.9 (11.0, 17.5)	15.4 (13.9, 21.7)	16.4 (13.7, 17.9)	0.032
REM, %	22.8 (15.2, 25.4)	20.5 (13.9, 22.6)	23.4 (17.1, 28.0)	0.045	22.6 (16.6, 25.3)	21.8 (16.7, 23.4)	24.2 (20.9, 26.9)	0.068
WASO, min	30.4 (21.8, 56.6)	34.0 (18.5, 81.6)	33.3 (23.8, 50.3)	0.836	22.5 (10.9, 48.1)	40.5 (10.1, 70.6)	26.0 (13.6, 42.1)	0.174
Sleep efficiency, %	87.8 (86.8, 94.6)	90.8 (80.5, 93.8)	91.8 (88.4, 93.4)	0.905	90.5 (86.9, 95.1)	91.1 (82.7, 96.2)	91.2 (88.4, 95.2)	0.174
Arousal index, /h	10.8 (9.1, 13.8)	17.5 (16.1, 20.5)[a]	14.5 (11.7, 17.0)[b]	0.014	10.3 (7.7, 14.8)	13.2 (9.8, 17.3)	11.1 (9.4, 14.2)	0.405
**Subjective sleep**								
Sleep latency, min	35.0 (10.0, 60.0)	20.0 (12.5, 30.0)	20.0 (12.5, 30.0)	0.127	30.0 (20.0, 30.0)	20.0 (10.0, 30.0)	20.0 (10.0, 30.0)	0.376
TST, min	405.0 (375.0, 427.5)	435.0 (397.5, 482.5)	435.0 (397.5, 483.0)	0.110	420.0 (360.0, 425.0)	430.0 (420.0, 480.0)	430.0 (420.0, 480.5)	0.767
Arousal, events	2.0 (1.0, 3.0)	2.5 (1.0, 4.3)	2.5 (1.0, 4.3)	0.368	3.0 (1.8, 3.3)	2.0 (1.0, 3.0)	2.0 (1.0, 3.0)	0.142
**Heart rate**								
NREM heart rate, beats/min	60.7 (56.8, 67.5)	77.1 (72.3, 88.3)[a]	78.6 (73.4, 86.9)[a]	0.007	58.5 (43.8, 63.1)	75.0 (71.6, 82.0)[a]	81.2 (75.4, 86.4)[a]	<0.001
REM heart rate, beats/min	62.0 (58.9, 68.5)	75.0 (71.3, 88.2)[a]	76.0 (72.9, 86.1)[a]	0.008	60.0 (46.2, 66.8)	76.2 (71.9, 82.6)[a]	78.7 (75.9, 83.2)[a]	<0.001

Values represent median (interquartile range). TST, total sleep time; NREM 1-3, non-rapid eye movement sleep stage 1-3; REM, rapid eye movement sleep; WASO, wake time after sleep onset. [a]$p < 0.05$ comparing with 500 m within the same sex; [b]$p < 0.05$ comparing with 3270 m 1st night within the same sex.

Table S2. Daytime evaluations in women and men at 500 m and 3270 m

	Men				Women			
	**500 m**	**3270 m 1st day**	**3270 m 2nd day**	**ANOVA, P value**	**500 m**	**3270 m 1st day**	**3270 m 2nd day**	**ANOVA, P value**
**AMS**								

AMS, score		1.0 (0, 2.8)	0 (0, 0.8)			3.0 (0.3, 4.0)	1.0 (0, 3)	
AMS, n (%)		1 (10.0)	1 (10.0)			7 (38.9)	4 (25.0)	
**PVT**								
Mean RT, ms	355 (348, 389)	362 (336, 391)	342 (338, 369)[*]	0.368	353 (338, 370)	386 (357, 405)[a]	389 (362, 419)[a]	0.001
Lapses, events	6.0 (2.0, 9.0)	5.0 (1.5, 7.0)	3.0 (1.0, 4.5)[*]	0.163	4.5 (1.3, 5.8)	6.0 (3.3, 10.0)	7.0 (4.0, 12.0)	0.125
Fastest 10% RT, ms	277 (253, 302)	271 (256, 306)	273 (250, 281)[*]	0.565	272 (263, 293)	284 (274, 306)	293 (273, 310)[a]	0.028
Slowest 10% RT, ms	520 (477, 704)	544(465, 688)	502 (459, 571)	0.565	501 (462, 537)	541 (500, 582)	502 (459, 571)	0.144
**Arterial blood analysis**								
pH	7.40 (7.39, 7.43)	7.45 (7.44, 7.47)[a]	7.46 (7.41, 7.48)[a]	0.005	7.41 (7.41, 7.42)	7.45 (7.43, 7.47)[a]	7.47 (7.45, 7.48)[a]	0.001
$PaCO_2$, mmHg	39.2 (35.2, 43.3)	35.5 (33.8, 39.3)	37.0 (33.5, 38.2) [*]	0.206	35.9 (34.4, 41.0)	34.0 (30.0, 38.0)	32.0 (28.5, 32.8)[a b]	0.005
PaO_2, mmHg	86.6 (81.6, 94.6)	56.0 (49.0, 60.3)[a*]	53.0 (49.5, 59.1)[a*]	0.001	90.3 (83.1, 95.9)	62.0 (55.0, 64.0)[a]	60.5 (57.5, 64.8)[a]	<0.001
HCO_3^-, mmol/L	24.8 (23.9, 26.0)[*]	25.1 (23.8, 26.5)	25.1 (23.8, 27.1)[*]	0.325	22.1 (21.8, 23.5)	24.2 (22.6, 25.1)	23.7 (20.5, 24.5)	0.052
SaO_2, %	97.8 (96.1, 98.0)	90.0 (85.5, 92.0)[a]	90.4 (87.5, 91.0)[a*]	0.001	97.8 (97.0, 98.0)	92.0 (88.0, 93.0)[a]	92.0 (92.0, 93.8)[a]	<0.001
**Venous blood analysis**								
Red blood cell, ^10^{12}/L	5.2 (5.0, 5.4)[*]	5.5 (5.1, 5.7)[*]	5.3 (5.2, 5.7)[*]	0.895	4.5 (4.2, 4.9)	4.6 (4.4, 4.7)[a]	4.8 (4.5, 5.1)[a]	0.012
Hemoglobin, g/L	157.0 (153.0, 162.5)[*]	162.9 (154.6, 167.0)[*]	159.0(155.0, 167.0)[*]	0.955	137.5 (132.5, 140.3)	137.6 (131.7, 142.0)	144.5 (134.8, 148.3)	0.068
Hematocrit, L/ L	0.45 (0.44, 0.47)[*]	0.48 (0.45, 0.50)[a*]	0.47 (0.46, 0.51)[a*]	0.045	0.39 (0.38, 0.41)	0.42 (0.39, 0.42) [a]	0.43 (0.41, 0.46)[a]	0.001
White blood cell, ^10^9/L	5.5 (4.9, 6.4)	6.9 (5.8, 8.5)	5.6 (5.0, 8.0)	0.102	5.6 (4.2, 6.4)	6.6 (5.2, 8.4)[a]	6.7 (5.1, 7.7)[a]	<0.001
Platelet, ^10^9/L	237.0 (206.8, 319.5)	244.0 (213.8, 272.8)	261.0 (238.0, 312.0)	0.396	228.5 (174.5, 254.5)	236.0 (191.8, 261.5)[a]	237.5 (197.3, 306.5)[a]	0.017

Values represent median (interquartile range). AMS, acute mountain sickness; PVT, psychomotor vigilance test; RT, reaction time. $PaCO_2$, partial pressure of carbon dioxide in artery; PaO_2, partial pressure of oxygen in artery; HCO_3^-, Bicarbonate Radical; SaO_2, oxygen saturation in artery. [a]$p < 0.05$ comparing with 500m within the same sex; [b]$p < 0.05$ comparing with 3270 m 1st night within the same sex; [*]$p < 0.05$ men vs. women at identical altitude and day.

Table S3 Univariate and multivariate generalized least square regression analysis models investigating predictors for AMS score and PVT

AMS severity, defined by the Lake Louise questionnaire				
	Univariate analysis		Multivariate analysis	
**Independent variables**	**Coefficient (95% CI)**	**P value**	**Coefficient (95% CI)**	**P value**
Age, years	-0.02 (-0.34, 0.31)	0.923		
BMI, kg/m^2	-0.09 (-0.29, 0.10)	0.352		
Sex (women vs. men)	1.21 (-0.02, 2.45)	0.054	1.16 (-0.07, 2.38)	0.060
AHI, /h	0.01(-0.05, 0.04)	0.860		
Central AHI, /h	-0.01 (-0.09, 0.09)	0.917		
Mean oxygen saturation, %	-0.03 (-0.16, -0.10)	0.606		
Total sleep time, min	-0.01 (-0.02, 0.01)	0.293		
Sleep efficiency, %	-0.05 (-0.12, 0.02)	0.183	-0.03 (-0.10, 0.04)	0.442
Arousal index, /h	-0.03 (-0.16, 0.10)	0.640		
Days at high altitude (2nd vs.1st)	-1.00 (-2.21, 0.21)	0.101	-0.89(-2.10, 0.32)	0.145
**PVT reaction time, 1/ms**				
	Univariate analysis		Multivariate analysis	
**Independent variables**	**Coefficient (95% CI)**	**P value**	**Coefficient (95% CI)**	**P value**
Age, years	0.007 (-0.017, 0.030)	0.575		
BMI, kg/m^2	-0.002 (-0.019, 0.015)	0.803		
Sex (men vs. women)	-0.076 (-0.201, 0.049)	0.198	-0.078 (-0.200, 0.043)	0.204
Total sleep time, min	-0.001 (-0.002, 0.001)	0.208		
NREM 3, %	-0.006 (-0.019, 0.007)	0.340		
Sleep efficiency, %	-0.001 (-0.011, 0.009)	0.877		
Wake time after sleep time, min	0.001 (-0.002, 0.002)	0.967		
Mean oxygen saturation, %	0.008 (0.001, 0.016)	0.029		
AHI, /h	-0.003 (-0.007, 0.002)	0.259		
Altitude (3270 m vs. 500 m)	0.143 (0.267, 0.019)	0.024	0.144(0.021, 0.268)	0.023
Days at high altitude (2nd vs.1st)	-0.052 (-0.182, 0.077)	0.422		

AMS, acute mountain sickness; PVT, psychomotor vigilance test; BMI, body mass index; NREM 3, non-rapid eye movement sleep stage 3; AHI, apnea hypopnea index.

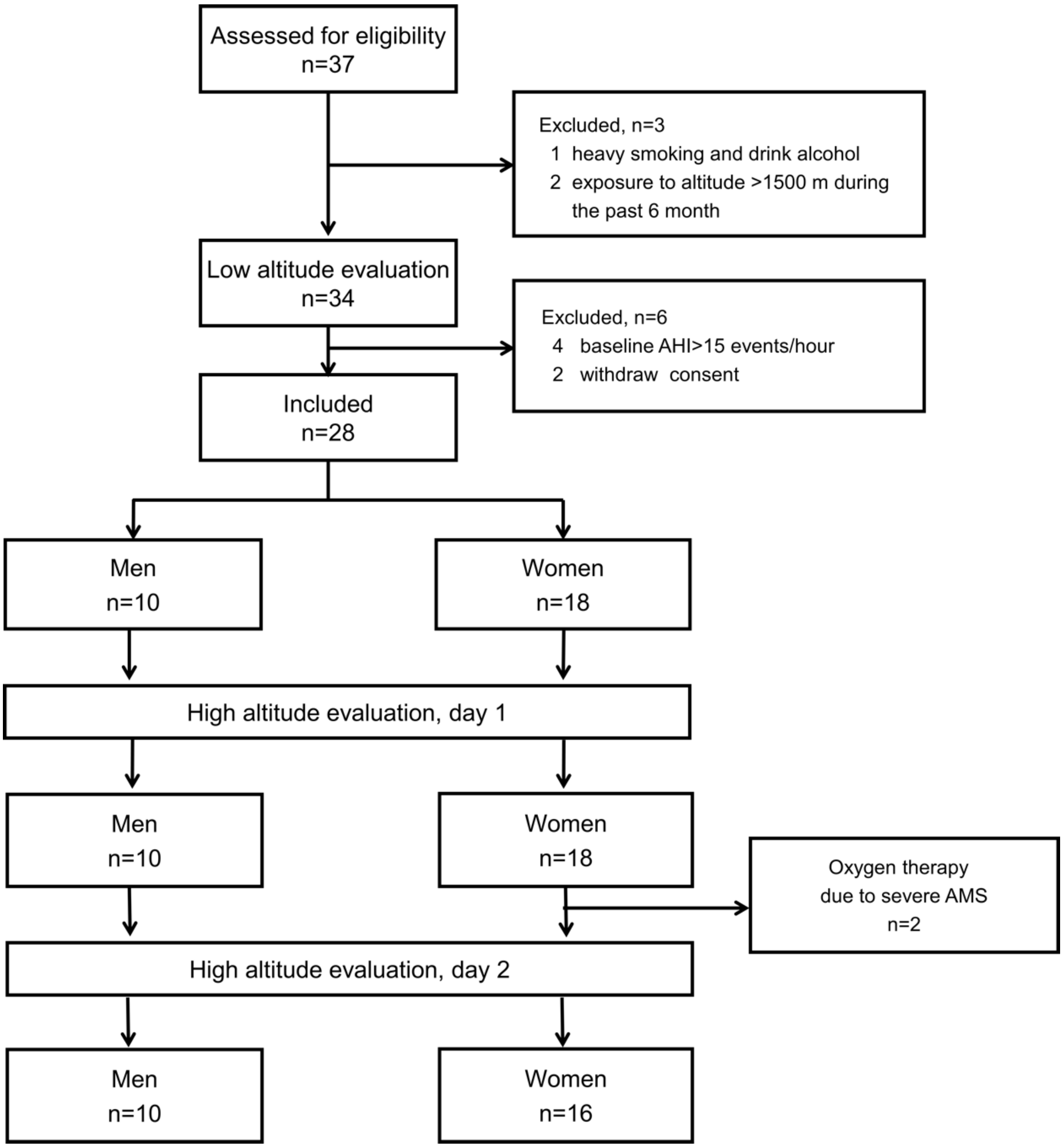

Figure S1. Flowchart.

Low altitude evaluations were performed at 500 m; high altitude evaluations at 3270 m.

AHI, apnea hypopnea index; AMS, acute mountain sickness

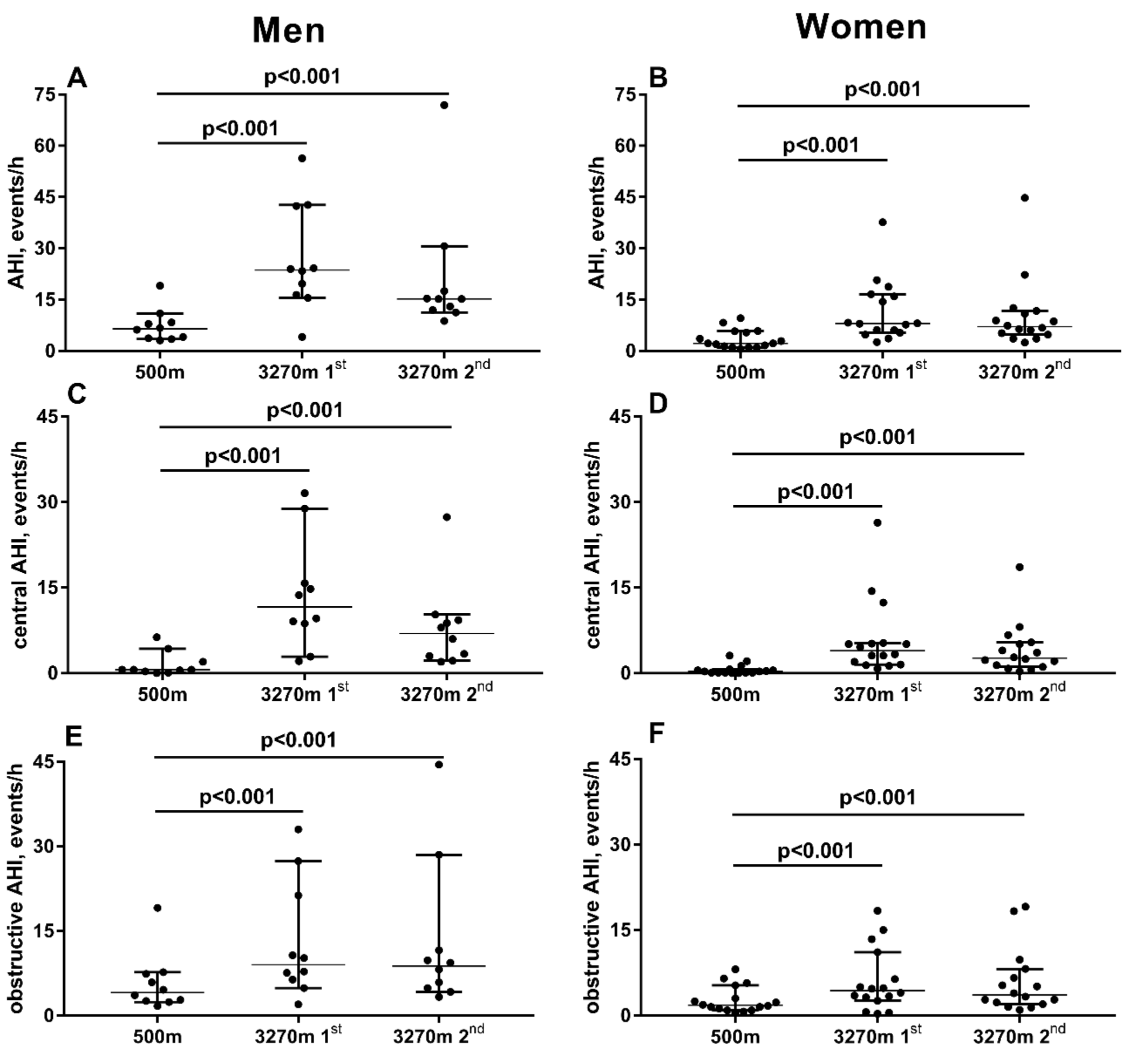

Figure S2 Individual values in total AHI (Panel A and B), central AHI (Panel C and D) and obstructive AHI (Panel E and F) at 500 m and 3270 m during the first and second night in men and women.

AHI, apnea hypopnea index.
